# Non-Syndromic Tooth Agenesis in Two Chinese Families Associated with Novel Missense Mutations in the TNF Domain of EDA (Ectodysplasin A)

**DOI:** 10.1371/journal.pone.0002396

**Published:** 2008-06-11

**Authors:** Shufeng Li, Jiahuang Li, Jian Cheng, Bingrong Zhou, Xin Tong, Xiangbai Dong, Zixing Wang, Qingang Hu, Meng Chen, Zi-Chun Hua

**Affiliations:** The State Key Laboratory of Pharmaceutical Biotechnology, Nanjing Stomatological Hospital, Nanjing University, Nanjing, People's Republic of China; National Institute of Neurological Disorders and Stroke, United States of America

## Abstract

Here we report two unrelated Chinese families with congenital missing teeth inherited in an X-linked manner. We mapped the affected locus to chromosome Xp11-Xq21 in one family. In the defined region, both families were found to have novel missense mutations in the ectodysplasin-A (*EDA*) gene. The mutation of c.947A>G caused the D316G substitution of the EDA protein. The mutation of c.1013C>T found in the other family resulted in the Thr to Met mutation at position 338 of EDA. The *EDA* gene has been reported responsible for X-linked hypohidrotic ectodermal dysplasia (XLHED) in humans characterized by impaired development of hair, eccrine sweat glands, and teeth. In contrast, all the affected individuals in the two families that we studied here had normal hair and skin. Structural analysis suggests that these two novel mutants may account for the milder phenotype by affecting the stability of EDA trimers. Our results indicate that these novel missense mutations in *EDA* are associated with the isolated tooth agenesis and provide preliminary explanation for the abnormal clinical phenotype at a molecular structural level.

## Introduction

Tooth development is a complex process with reciprocal interactions between the dental epithelium and mesenchyme; many transcription factors and signaling molecules are involved to guide this process. Tooth agenesis is the most common craniofacial malformation with patients missing one or more teeth. The term ‘hypodontia’ is defined as the congenital absence of fewer than 6 teeth, whereas ‘oligodontia’ designates the congenital absence of 6 or more permanent teeth and the complete absence of teeth is defined as ‘anodontia’. Tooth agenesis can occur in an isolated fashion, or as part of a syndrome. Affected members within a family often exhibit significant variability with regard to the location, symmetry, and number of teeth involved.

To date, mutations in two transcription factor genes, *PAX9* (14q12–13) and *MSX1* (4p16.1), have been shown as the major causes of non-syndromic oligodontia. *MSX1* is a member of the homeobox gene family which contains a homeodomain. MSX1 is strongly expressed in the dental mesenchyme and is eliminated from the dental epithelia during the bud, cap, and bell stages of tooth development [1]. Mutations in *MSX1* coding regions cause human tooth agenesis of various types of teeth, preferentially premolars [2]. *PAX9* belongs to the *PAX* gene family, which encodes a group of transcription factors that play a role in early development. PAX proteins are defined by the presence of a DNA-binding domain, the ‘paired domain’, which makes sequence-specific contact with DNA. PAX9 is expressed in the neural-crest-derived mesenchyme of the maxillary and mandibular arches, and contributes to palate and tooth formation [3]. Mutations in *PAX9* coding regions or a *PAX9* deletion causes preferential tooth agenesis of molars [4]–[9]. In addition to *PAX9* and *MSX1*, *AXIN2*, which encodes a Wnt-signaling regulator, is reported to associate with oligodontia and colorectal neoplasia [10]–[11].

Among the missing teeth syndrome, oligodontia often occurs with other ectodermal dysplasias, including nail dysplasia, dry skin, fine hair, and sweating defects [12]–[14]. There are more than 49 syndromes that are associated with tooth agenesis [15]. X-linked hypohidrotic or anhidrotic ectodermal dysplasia (XLHED, MIM 305100) is the most common form of ectodermal dysplasia and is characterized by sparse hair, eye lashes and brow, abnormal or missing teeth and inability to sweat due to the lack of sweat glands, a major disability in hot climates. The facial appearance of XLHED consists of a saddle-nose, frontal bossing and thick lips. Atopic dermatitis and bronchial asthma are frequent complications [16]. If unrecognized, XLHED is one of the causes of fevers of unknown origin, repeated bronchitis, and sudden death during infancy and early childhood. Affected males present most or all of these typical features. In female carriers, the severity of the disorder varies considerably, but most of them have mild to moderate manifestations of these typical features, ranging from none to some degree of hypodontia, hypotrichosis, and hypohidrosis.

Mutations of the ectodysplasin A (*EDA*) gene are responsible for this disorder [17]–[20]. EDA is a 391 amino acid transmembrane protein with a C-terminal TNF domain [19], [21], [22] which involves in the early epithelial–mesenchymal interaction that regulates ectodermal appendage formation [21]. It is a ligand for a death-domain- containing receptor called the ectodysplasin-A receptor (EDAR). The ligand receptor pair signals through an adaptor molecule, called ectodysplasin-A receptor associated death domain (EDARADD), to the nuclear factor-kappa B (NF-κB) pathway to promote cellular survival [23]–[25]. Mutations in EDAR produce an identical phenotype to the loss of function of EDA [19]. EDA/EDAR interaction was also reported to regulate mouse tooth development [26].

This study describes two unrelated Chinese families affected by non-syndromic tooth agenesis in an X-linked manner. The objective of the present study was to identify the mutation responsible for the familial tooth agenesis in kindred and to identify genotype/phenotype correlations that could improve the understanding of abnormal and arrested tooth formation.

## Results

### Clinical Examination

**Figure 1:**
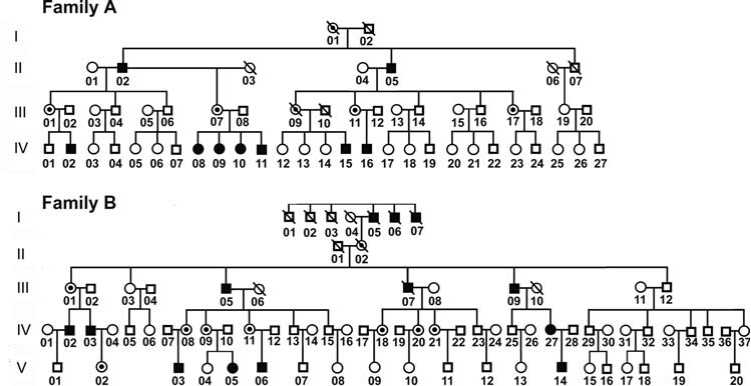
Pedigree structure of the two Chinese families with tooth agenesis. Affection status for pedigrees (females in circles, males in squares) is shown: closed symbols, affected; open symbols, unaffected.

The pedigrees are shown in Fig. 1. Both the families showed an apparent X-linked dominant form of tooth agenesis. Family A comprises 56 members spanning four generations, 9 members in this family show congenital tooth agenesis (male 6, female 3). Family B consists of five generations including 13 members with congenital tooth agenesis (male 11, female 2).

**Figure 2:**
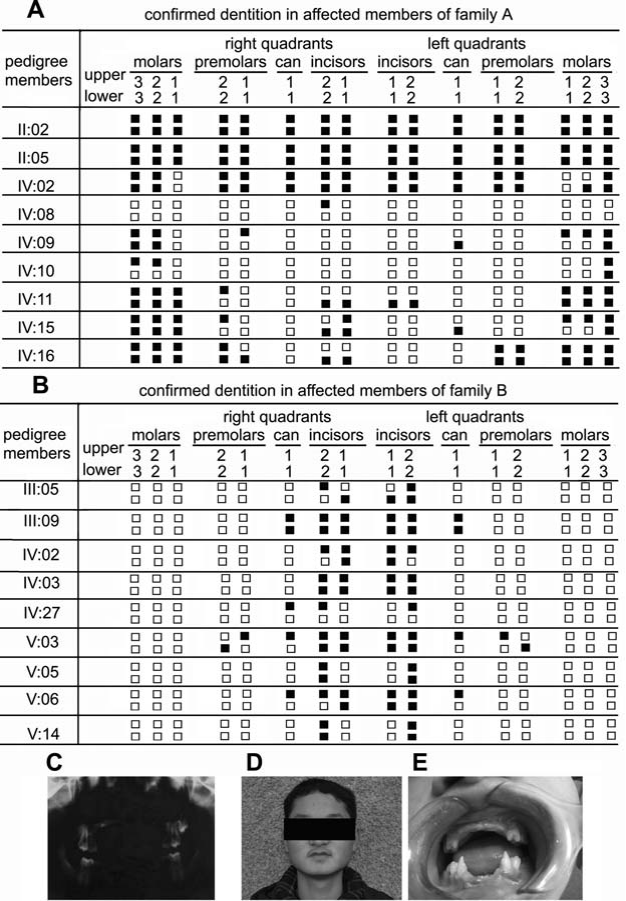
Clinical evaluations. (A) and (B). Synopsis of the permanent dentition in affected members of Family A and Family B. Closed squares represent absent teeth. (C). Panoramic radiograph of a proband (IV:02) in Family A at age 14. He had up left permanent second molar, four permanent first molars and four milk second molars. (D) and (E): Clinical appearance of V:03 (Family B), at age 18, showing absence of all the incisors, two up cans and four premolars.

In both families, the manifestation of tooth agenesis is not uniform (Fig. 2). In Family A, the hypodontia involved all classes of teeth. However, in Family B, all the affected members predominantly lacked incisor teeth but had all the permanent molars. The phenotype in Family A was more severe in terms of the numbers of missing teeth, *i.e*., with the proband lacking all permanent premolars, cans, incisors, as well as all the third molars and three second molars. Phenotypic characteristics of scalp and body hair, skin, nails and ability to sweat were examined in all individuals of both families. All the individuals had normal sweating and had no complaints about intolerance to heat, and their facial features, skin, and nails all appeared normal.

### Positional cloning of the oligodontia gene

**Figure 3:**
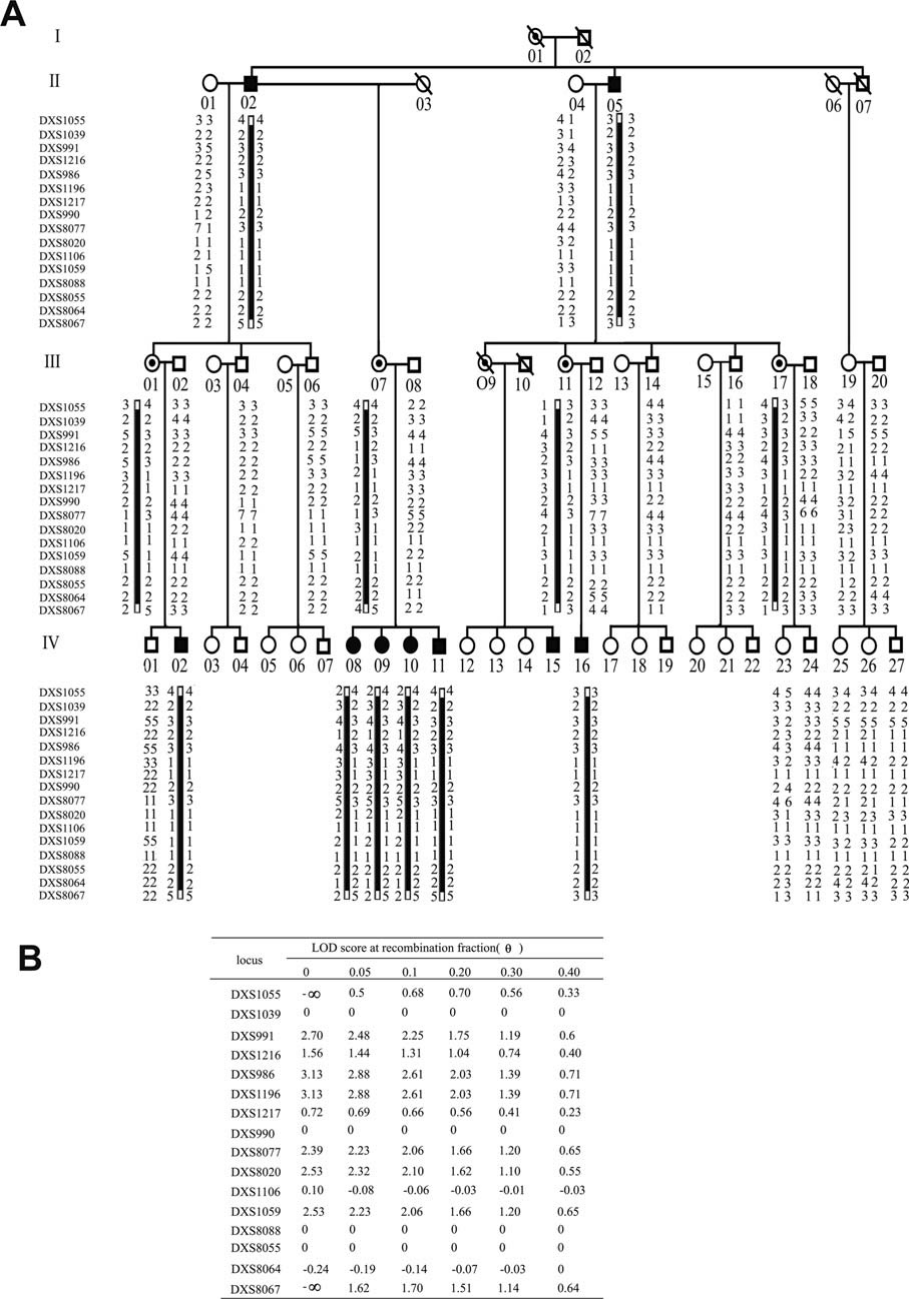
Linkage analysis for Family A. (A). Haplotype analysis in the Family A. Open symbols represent the unaffected individuals, closed symbols represent the affected individuals, squares indicate men, and circles indicate women. The closed bars indicate the fourteen contiguous-marker disease-linked haplotypes shared by all patients and female carriers. (B).The Lod Score obtained from the analysis of the pedigree of Family A with X-linked microsatellite markers around the centromere.

By haplotype analysis of the pedigree of Family A (Fig. 3A), the affected locus was confined to the region between DXS1039 and DXS8064. In the two-point linkage analysis, two loci in this region, DXS1196 and DXS986, both gave the highest two-point LOD value of 3.13 (Fig. 3B), strongly suggesting that the disease gene associated with tooth agenesis is closely linked to the two markers.

**Figure 4:**
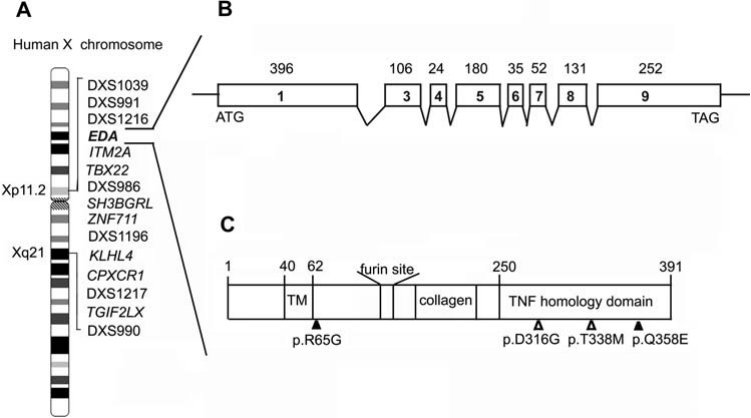
Candidate genes identified in the critical region in the Xp11-Xq 21. (A). The gene order for this region as obtained from NCBI (NCBI Map Viewer, human Build 36.2). (B). The *EDA* gene structure. The numbered boxes represent exons and the connecting lines represent intronic regions. (C). The structural feature of protein product of *EDA*. The start and stop codons are indicated by “ATG” and “TAG.” The transmembrane domain (“TM”), furin recognition site, collagen domain, and the TNF homology domain are shown. The relative positions of the mutations identified in this article are indicated by open triangles. Mutations found in previous studies associated with X-linked hypodontia, are also indicated in this diagram by closed triangles.

In this critical region, the candidate genes for the oligodontia locus include those encoding for transcription factors or proteins involved in signal transduction. The relative positions of these genes are shown in Fig. 4A. We therefore proceeded to screen for mutations in these genes. By directly sequencing all exons and flanking splice junctions of the candidate genes, *ITM2A, TBX22, SH3BGRL, ZNF711, KLHL4, CPXCR1* and *TGIF2LX* were ruled out because no mutation was detected (data not shown). Our best candidate pointed to *EDA*. The *EDA* gene structure is shown in Fig. 4B.

### Mutation analysis

**Figure 5:**
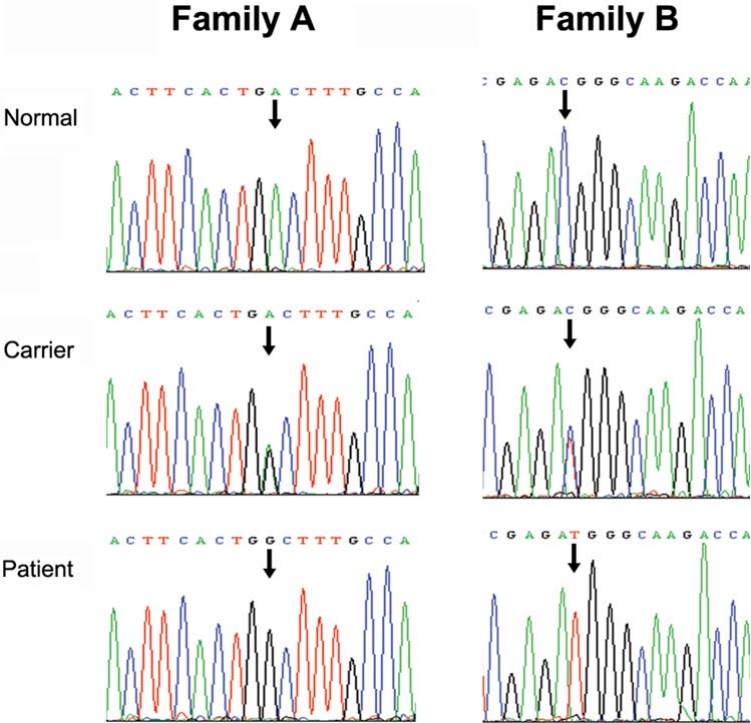
DNA sequencing chromatograms showing the mutations of affected members in Family A and Family B. In Family A, male patients show c.947A>G transition that is predicted to result in a p.D316G substitution in EDA and female carriers have A/G heterozygosity at the same position. In Family B, male patients show c.1013C>T transition that is predicted to result in a p.T338M substitution in EDA and female carriers are C/T heterozygous at the same position. Patient: male patients (hemizygous), carrier: female carriers (heterozygous), normal: normal individuals (no mutation).

To identify possible mutations in the *EDA* gene, we first sequenced all eight exons coding for EDA in two affected males and two female carriers in Family A. We found a novel missense mutation c.947A>G in exon 9 of *EDA*, and found the mutation segregating with affected or carrier status in the other family members (Fig. 5). This mutation resulted in the non-synonymous substitution of aspartic acid for glycine at amino acid residue position 316 (p.D316G) in the TNF domain of EDA protein (Fig. 4C). Exon 9 of *EDA* was also sequenced from 300 unrelated normal Chinese individuals with the same Han ethnic background, 150 females and 150 males, without detecting any guanine at the c.947 base position of *EDA* gene allele.

Though the phenotype of hypodontia in Family B was milder than Family A, the hypodontia in Family B was inherited as the same X-linked dominant trait as in Family A. Therefore we also selected *EDA* as a candidate gene in Family B and directly examined Family B for possible mutations in *EDA*. After mutation screening of eight exons of *EDA* gene in Family B, a c.1013C>T transition mutation of *EDA* gene was observed (Fig. 5). The c.1013C>T transition resulted in p.T338M substitution in the TNF domain of the EDA protein (Fig. 4C). The c.1013C>T mutant allele was present in all affected males, and in affected and obligate carrier females, but in none of the other individuals in Family B. The c.1013C>T nucleotide substitution was also not found in any of 300 control individuals of the same ethnic background, which strongly suggests that this is the causative mutation in this family.

In both families, the male individuals bearing the mutant gene were associated with complete penetrance, however, in female heterozygotes incomplete penetrance was observed. In Family A, female carries IV:08, IV:09 and IV:10 showed hypodontia, but the other carriers displayed normal teeth (Fig. 1). Among female carriers in Family B, only individuals IV:27 and V:05 showed missing teeth, while the other female carriers showed normal teeth. The cause of this phenomenon is unclear; however, it may have resulted from the differential pattern of X-chromosome inactivation between the symptomatic carriers and the non-symptomatic carriers.

### Molecular Modeling

The *EDA* gene product is a type-II transmembrane protein with a small N-terminal intracellular domain followed by a larger C-terminal extracellular domain. The C-terminal extracellular domain contains a collagen-like repeat domain and a tumor necrosis factor (TNF) domain [21] (Fig. 4C). The TNF domain has been shown to form homotrimers which are believed to be required for receptor interactions [27]. The HED-causing mutations in the TNF domain which affect the function of EDA have been previously analyzed: most mutations (His252Leu, Gly291Trp, Gly291Arg, Gly299Ser, Tyr320Cys, and Ala349Asp) are likely to affect the overall structure of EDA, and some mutations (Tyr343Cys, Ser374Arg, Thr378Pro, and Thr378Met) alter the receptor binding site [27]. However, Asp316Gly and Thr338Met mutations have not been reported previously.

**Figure 6:**
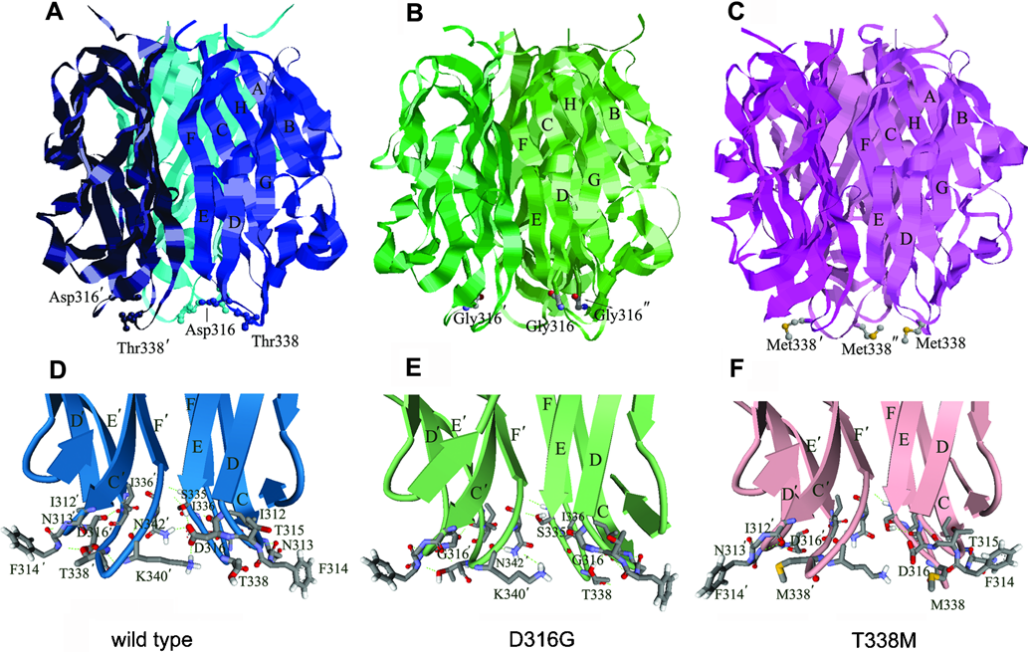
Structures of wild type, D316G and T338M EDA. (A)–(C). Locations of p.D316G or p.T338M mutation sites in the quaternary structure of the EDA homotrimers. The EDA trimers are shown as ribbon rendering with mutation residues rendered in stick and ball, beta strands and the mutated amino acids are labeled. (D)–(F). Close up views of the mutation sites of D316G, T338M and wild type EDA proteins. The amino acid backbones are represented by ribbons with arrows. Side chains have been omitted from most residues for clarity. Hydrogen bonds are indicated by dashed green lines.

Structural analysis of EDA protein showed that D316 and T338 were located at two adjacent loops at the bottom of the TNF domain (D316 at loop CD and T338 at loop EF respectively) (Fig. 6A–6C). Unlike the above mentioned HED-causing mutations, both p.D316G and p.T338M mutations were a distance away from the receptor-binding site, and thus are not likely required for the receptor binding. To understand the structural effects of these two point mutations, the detailed molecular modeling analysis of the interactions between mutated residues and their surrounding residues was performed and is presented below.

Residue Asp316, which is located at loop CD, interacted with the residues including Ser335, Ile336 and Thr338 in the adjacent loop EF via van der Waals force or hydrogen bonds. In addition, the side chain of Asp316 extended and formed salt bridges or hydrogen bonds with Lys340 or Asn342 from the neighboring monomer, thus taking part in subunit-subunit interactions (Fig. 6D). Replacing this residue by Gly abolished the contacts between the side chain of Asp316 and its surrounding residues and its neighboring monomer (Fig. 6E). This could reduce the inter-subunit interactions in this region and further affect the stability of the trimer. The mutation to Gly also decreased the hydrophilicity and negative charges of this site and may increase the flexibility of this region. Considering its role in interactions between loops, this Gly mutation may also affect the fluctuation of the adjacent loop EF.

Thr338, located in loop EF, could interact with the residues from loop CD, forming a hydrogen bond with the carbonyl oxygen atom of Ile312. In addition, backbone and side chain atoms of Thr338 could interact with Asn313, Thr315 and Asp316 via van der Waals force or hydrogen bond (Fig. 6D). The replacement of hydrophilic Thr with hydrophobic Met increased the hydrophobicity at this site. The absence of Thr338 in p.T338M mutant would cause a decrease in the stability of this region. Meanwhile, the large hydrophobic side chain of Met338 in the mutant might make hydrophobic and van der Waals interactions with Asn313 and Phe314 from neighbor loop CD and thus may contribute to the conformational rearrangement surrounding the residues. As a consequence, it could affect the conformation of Asp316 side chain and in turn disrupt the inter-subunit interactions (Fig. 6F). These results by molecular modeling suggest that replacing hydrophobic residue at this site would not only affect the stability of loop EF but also affect the stability of the nearby loop CD.

In conclusion, this molecular modeling analysis suggests that although Asp316 and Thr338 are located at different sites in the primary sequence, they are spatially close and might interact with each other; the mutation in either site could affect the stability of its adjacent loop. It is possible that p.D316G and p.T338M might have similar impacts on the structure of EDA by interacting between each other. As strands E, F and C connected by CD and EF loops are directly involved in the inter-subunits interactions and Asp316 and its neighboring residues are directly involved in monomer-monomer interactions, it could be expected that significant variations occurring at these mutated sites would affect the stability of EDA trimer.

## Discussion

In this study, we identify two novel mutations in the *EDA* gene in two independent Chinese families with isolated tooth agenesis. Both mutations are predicted to result in changes in single amino acid residues in the TNF domain of the protein: p.D316G and p.T338M. These mutations of p.D316G and p.T338M in EDA and their effects on the phenotype change have not been reported previously.

Mutations in ectodysplasin A have been previously identified as the cause of X-linked hypohidrotic ectodermal dysplasia (XLHED) (OMIM 305100). So far, only two articles reported that mutations in the *EDA* gene resulted in unique hypodontia phenotype rather than the full XLHED phenotype [28]–[29]. In that study the test group one was a Mongolian family with a missense mutation (p.R65G) in the juxtamembrane region of EDA [28]. Affected members in this Mongolian family did not show other XLHED characteristics, except hypodontia. The patterns of missing teeth were similar to the Family A in our study; the hypodontia occurring in both the Mongolian family and Family A involved all classes of teeth. Another previously reported study of an Indian family found a missense mutation (p.Q358E) in the EDA TNF domain. The phenotype of this Indian family most resembled the Family B of our study [29]. Both Family B and the Indian family show incisor hypodontia and the symptom is milder than that observed in Family A and the Mongolian family. It seems that different mutation sites in EDA caused the difference in missing teeth patterns. Interestingly, a similar finding was also observed caused by the mutations in the *PAX9* gene. Lammi et al. reported a missense mutation in *PAX9* in a family with oligodontia phenotype that affected all tooth groups [7]. In another report, Kapadia et al. showed Ile87Phe mutation in PAX9 in another family only affected posterior teeth, primarily the molars [30].

In this report, we describe the identification of two novel missense mutations in the TNF domain of EDA. Both mutations associated with only tooth agenesis without causing other abnormalities. Unlike other reported HED-causing mutations in the TNF domain which were expected to affect the receptor binding site or overall structure of the EDA protein, molecular modeling suggests that these two mutations only minimally affect the stability of EDA trimer. Therefore, we presume that the human phenotype associated with the novel mutations could be caused by the decrease in the stability of the EDA protein. The phenotype of isolated tooth agenesis of the two families may be a milder clinical subtype of X-linked HED. Similar to p.D316G and p.T338M, p.Q358E is also located on the outer surface of the EDA protein. Tarpey et al [29] suggested that Q358E partially disrupts the interaction of the EDA homotrimers, thus resulting in the inability of EDA to interact with its target receptors. Until now, there have been four EDA mutations, p.R65G, p.Q358E, p.D316G and p.T338M that have been reported associated with unique tooth agenesis. p.Q358E, p.D316G and p.T338M are all in the TNF domain. These three mutants seem to share similar consequences: they all disrupt the interactions in the homotrimer and therefore result in the inability of EDA to interact with its target receptors. One unique feature among these mutants is that D316G is directly involved in interactions of the monomers, while the other two mutations indirectly affect the interactions of the EDA homotrimers. This difference may account for the phenotype of p.D316G being more severe than that of p.T338M and the phenotype of p.T338M is very similar to that of p.Q358E. In the p.R65G mutant, the substitution occurs on the edge of the transmembrane domain of the EDA protein and its effect on EDA function is still not clear.

Here we identify novel mutations in the *EDA* gene that are involved in the X-linked isolated hypodontia and provide an explanation for the clinical phenotype at the molecular structural level. This is an initial step in explaining the pathogenic mechanisms underlying EDA-mediated tooth agenesis. Further in vivo expression and functional characterization of the mutated protein might present more direct explanations for how the malfunctions in EDA protein caused by the identified missense mutations disrupted the tooth development.

## Materials and Methods

### Pedigree, diagnosis, and DNA collection

The research project was submitted and approved by the Institutional Review Board (IRB) of Nanjing University. Detailed histories and pedigree information about the affected two families, which were designated Family A and Family B (Fig. 1), were obtained and confirmed through personal interviews with family members. The oral status of each individual was scored as affected or unaffected by personal examination and/or by review of dental records and X-ray films. After consents were obtained from all participating individuals, venous blood samples were collected and genomic DNA was isolated with the QIAmp Blood kit (Qiagen, Germany). In addition to these two families, 300 normal unrelated individuals of the same ethnic background (150 male and 150 female) were recruited as controls.

### Microsatellite genotyping

Since the pedigrees showed the X-linked inheritance mode, 48 polymorphic microsatellite genetic markers, spanning the entire X chromosome at the average interval of 5 cM (Linkage Mapping Set-HD5 kit , PE Biosystems, Foster City, CA, USA), were selected to scan the genomic DNA extracted from peripheral blood leukocyte of Family A members. Two-point linkage analysis was performed, using parametric method with the MLINK software of the Linkage Analysis Package version 5.2.

### Mutation analysis

By haplotype analysis of the pedigree, we confirmed that *EDA* is our best candidate gene. Previously designed primers flanking the coding regions of the eight exons of *EDA* were used to amplify the genomic DNA by polymerase chain reaction [22]. All eight exons and their flanking splice junctions of *EDA* were directly sequenced by using an ABI-3100 sequencer.

### Homology modeling

The X-ray structure of the TNF domain of EDA was obtained from PDB (ID:1RJ7). The monomer structures of D316G and T338M TNF domain of EDA were built by MODELLER8v2 program (http://salilab.org/modeller/)[31]. Trimers of EDA mutants were built using Insight II (Accelrys, San Diego, CA, USA).
